# First Complete Genome Sequence of *Marinilactibacillus* *piezotolerans* Strain 15R, a Marine Lactobacillus Isolated from Coal-Bearing Sediment 2.0 Kilometers below the Seafloor, Determined by PacBio Single-Molecule Real-Time Technology

**DOI:** 10.1128/genomeA.01625-16

**Published:** 2017-02-16

**Authors:** Yuli Wei, Junwei Cao, Jiasong Fang, Chiaki Kato, Weicheng Cui

**Affiliations:** a Shanghai Engineering Research Center of Hadal Science and Technology, College of Marine Sciences, Shanghai Ocean University, Shanghai, China; b College of Natural and Computational Sciences, Hawaii Pacific University, Kaneohe, Hawaii, USA; c Department of Marine Biodiversity Research, Japan Agency for Marine-Earth Science and Technology, Yokosuka, Japan

## Abstract

*Marinilactibacillus piezotolerans* strain 15R is a facultatively anaerobic heterotrophic lactobacillus isolated from deep marine subsurface sediment nearly 2 km below the seafloor in the northwestern Pacific. We report here the first whole-genome sequence of strain 15R. The identified genome sequence has 2,767,908 bp, 35.4% G+C content, and predicted 2,552 candidate protein-coding sequences, with no identified plasmids.

## GENOME ANNOUNCEMENT

*Marinilactibacillus piezotolerans* strain 15R can ferment a wide range of organic substrates to produce lactic acid as the main end product of metabolism (1). *Marinilactibacillus piezotolerans* could play a significant role in the transformation of organic matter in deep marine sediments (1). However, little is known about their origin and adaptation in the deep biosphere. At present, the genus of *Marinilactibacillus* comprises only two species, *Marinilactibacillus piezotolerans* from deep subseafloor sediment of the Nankai Trough (1), and* Marinilactibacillus psychrotolerans* from dead and living marine organisms in temperate and subtropical areas of Japan (2).

*Marinilactibacillus piezotolerans* strain 15R is a facultatively anaerobic heterotrophic piezotolerant bacterium, isolated from deep marine subsurface sediment nearly 2 km below the seafloor from the northwestern Pacific (unpublished data). Strain 15R was cultivated in 100 ml of marine broth 2216 at 35°C and 0.1 MPa for 24 h, and genomic DNA was extracted and purified using Qiagen Genomic-tip 500/G kit (Qiagen, Düsseldorf, Germany), as per the manufacturer’s protocol. DNAs were sheared by utilizing g-Tubes (Covaris, Inc., Woburn, MA). The 8- to 12-kbp sheared products were further size selected utilizing 0.45× AMPure beads. The sheared DNAs were used to generate large SMRTbell libraries using the standard library protocols of the Pacific Biosciences DNA template preparation kit 4.0 (Pacific Biosciences, Menlo Park, CA, USA). Sequence reads were filtered and assembled *de novo* utilizing the PacBio Hierarchical Genome Assembly Process version 3 (HGAP3) (3). The PacBio RSII sequencing platform (Pacific Biosciences) was employed to obtain the sequence reads in the Chinese National Human Genome Center in Shanghai, China. The completed genome sequence of* Marinilactibacillus piezotolerans* 15R comprises a 2,767,908-bp circular chromosome (439-fold coverage and 35.4% G+C content).

The genome was annotated using the NCBI Prokaryotic Genome Annotation Pipeline (http://www.ncbi.nlm.nih.gov/genome/annotation_prok/). A total of 2,552 protein-coding sequences were identified, as well as 5, 4, and 4 (5S, 16S, and 23S, respectively) rRNA operons and 64 tRNA genes. Genome sequence analysis showed that the gene encoding aldolase and phosphofructokinase were found in *Marinilactibacillus piezotolerans* strain 15R. Lactobacilli were classed into a homofermentative group and heterofermentative group, according to a core genome phylogenetic tree (4). Aldolase and phosphofructokinase were generally present in homofermentative but not in heterofermentative lactobacilli (4), suggesting that *Marinilactibacillus piezotolerans* strain 15R is a homofermentative lactobacillus. Until now, only a few members of the genus *Marinilactibacillus* have been isolated from the marine environments. Therefore, this complete genome sequence will help us to understand its origin, adaptation, function, and metabolism in the deep biosphere.

### Accession number(s).

The complete genome sequence of *Marinilactibacillus piezotolerans* strain 15R has been deposited in GenBank under accession number CP017761.
